# Contribution of CFTR to Alveolar Fluid Clearance by Lipoxin A_**4**_ via PI3K/Akt Pathway in LPS-Induced Acute Lung Injury

**DOI:** 10.1155/2013/862628

**Published:** 2013-05-16

**Authors:** Yi Yang, Yang Cheng, Qing-Quan Lian, Li Yang, Wei Qi, De-Rong Wu, Xia Zheng, Yong-Jian Liu, Wen-Juan Li, Sheng-Wei Jin, Fang Gao Smith

**Affiliations:** ^1^Department of Anesthesia and Critical Care, Second Affiliated Hospital of Wenzhou Medical College, 109 Xueyuan Road, Wenzhou, Zhejiang 325027, China; ^2^Department of Pathophysiology, Wenzhou Medical College, Zhejiang 325027, China; ^3^Academic Department of Anesthesia, Critical Care, Pain and Resuscitation, Birmingham Heartlands Hospital, Heart of England NHS Foundation Trust, Birmingham B9 5SS, UK

## Abstract

The lipoxins are the first proresolution mediators to be recognized and described as the endogenous “braking signals” for inflammation. We evaluated the anti-inflammatory and proresolution bioactions of lipoxin A_4_ in our lipopolysaccharide (LPS-)induced lung injury model. We demonstrated that lipoxin A_4_ significantly improved histology of rat lungs and inhibited IL-6 and TNF-**α** in LPS-induced lung injury. In addition, lipoxin A_4_ increased alveolar fluid clearance (AFC) and the effect of lipoxin A_4_ on AFC was abolished by CFTR_inh-172_ (a specific inhibitor of CFTR). Moreover, lipoxin A_4_ could increase cystic fibrosis transmembrane conductance regulator (CFTR) protein expression *in vitro* and *in vivo*. In rat primary alveolar type II (ATII) cells, LPS decreased CFTR protein expression via activation of PI3K/Akt, and lipoxin A_4_ suppressed LPS-stimulated phosphorylation of Akt. These results showed that lipoxin A_4_ enhanced CFTR protein expression and increased AFC via PI3K/Akt pathway. Thus, lipoxin A_4_ may provide a potential therapeutic approach for acute lung injury.

## 1. Introduction

Acute lung injury (ALI) is a critical illness syndrome characterized by an increased permeability of the alveolar-capillary barrier resulting in impairment of alveolar fluid clearance (AFC). A major cause for development of ALI is sepsis, wherein Gram-negative bacteria are a prominent cause. Lipopolysaccharide (LPS), the outer membrane of Gram-negative bacteria, was the one of mainly pro-inflammatory reaction factor in lung injury, leading to neutrophil recruitment and pulmonary edema [1]. Alveolar fluid clearance in patients with ALI and the acute respiratory distress syndrome (ARDS) is impaired in the majority of patients, and maximal alveolar fluid clearance is associated with better clinical outcomes [2].

AFC depends on active ion transport, which leads to an osmotic gradient that drives the movement of fluid from the alveolar space back into the interstitium and eventually to the blood circulation [3]. The cystic fibrosis transmembrane conductance regulator (CFTR) is a chloride channel expressed in both alveolar type I (ATI) and alveolar type II (ATII) cells [4–6]. More recent studies have shown that upregulation of alveolar CFTR function in rat and mouse lungs speeds clearance of excess fluid from the airspace and that CFTRs effect on active Na^+^ transport requires the beta-adrenergic receptors, which can be blocked by nonspecific chloride channel inhibitors [7]. In addition, absence or inhibition of CFTR leads to lack of increase in alveolar liquid clearance in response to beta adrenergic agonist [8, 9]. This evidences suggests that CFTR chloride channel contributes to AFC.

Although significant efforts have been made to pharmacologically upregulate alveolar fluid clearance to reverse the progression of lung injury, these approaches have not been proven effective. Previous studies have demonstrated that *β*-adrenergic receptor agonists, glucocorticoids, and several growth factors stimulated alveolar fluid clearance in animal models of ALI [10–12]. Based on *in vivo* and* in vitro* work, we demonstrated that salbutamol stimulated alveolar epithelial repair and reduced extravascular lung water [13, 14]. However, in a multicentre, randomized controlled trial, we found that intravenous salbutamol increased 28-day mortality in patients with ARDS [15]. Thus, new insights are needed to provide novel therapeutic approaches for ALI and ARDS.

The lipoxins are the first proresolution mediators to be recognized and described as the endogenous “braking signals” for inflammation [16]. Lipoxins elicit distinct antiinflammatory and proresolution bioactions, including inhibition of neutrophil functional responses, T-cell activation, and pro-inflammatory cytokines release [17]. We have previously demonstrated the biphasic role of lipoxin A_4_ on expression of cyclooxygenase-2 (COX-2) in LPS-stimulated lung fibroblasts and the therapeutic effect of lipoxin A_4_ in LPS-induced ALI [18, 19]. However, whether lipoxin A_4_ can increase AFC stimulated by LPS and, if so, what the underlying mechanisms are, remain unclear.

In the present study, we examined the effects of lipoxin A_4_ on AFC in LPS-induced ALI rats. Additionally, we also investigated its effects on the CFTR protein expression in the rat lungs and primary ATII cells. Finally, to gain a better understanding of the mechanisms, we investigated the signaling pathways which regulated the effects of lipoxin A_4_.

## 2. Materials and Methods

### 2.1. Reagents

Lipoxin A_4_, from Cayman Chemical Company, was stored at −80°C before use. LPS (*E. coil* serotype 055: B5), Evan's blue, CFTR_inh-172_, LY294002, U0126, and deoxyribonuclease I were purchased from Sigma. ELISA kits of IL-6 and TNF-*α* were purchased from R&D Systems. DMEM, FCS, and Trypsin EDTA were purchased from Gibco. Penicillin and streptomycin in saline citrate buffer were from Invitrogen. Anti-CFTR and anti-*β*-actin were purchased from Santa Cruz. Anti-phospho-ERK and anti-phospho-Akt were purchased from Cell Signaling Technology.

### 2.2. Animal and Preparation

Male Sprague-Dawley rats (200–300 g) were purchased from Shanghai SLAC Laboratory Animal Co. Ltd. All Animal experiments were permitted by the Animal Care Committee of Wenzhou Medical College.

The rats were divided into four groups (*n* = 10): (1) control group, in which the rats were treated with 0.1% ethanol (vehicle for lipoxin A_4_, 5 mL/kg, iv) 6 h after they were treated with 0.9% saline (vehicle for LPS, 4 mL/kg, iv); (2) LPS group was identical to the control group except that LPS (20 mg/kg, iv) was administered instead of its vehicle; (3) lipoxin A_4_ treatment group was identical to the LPS group except that lipoxin A_4_ (2 *μ*g/kg, iv) was administered instead of its vehicle; (4) CFTR_inh-172_ group was identical to the lipoxin A_4_ treatment group, but CFTR_inh-172_ (1 uM, tracheal instillation) was administered after lipoxin A_4_.

For the experiment, different concentrations of LPS including 10 mg/kg, 15 mg/kg, 20 mg/kg and lipoxin A_4_ including 1 *μ*g/kg, 1.5 *μ*g/kg, 2 *μ*g/kg, 2.5 *μ*g/kg, 3 *μ*g/kg were treated to measure the AFC.

### 2.3. Measurement of AFC in Live Rats

Preparation of the alveolar instillate was as follows: a 5% albumin instillate solution was prepared by dissolving 50 mg/mL of bovine serum albumin in modified lactated Ringers solution: 137 mM NaCl, 4.67 mM KCl, 1.82 mM CaCl_2_  ∗ 2H_2_O, 1.25 Mm MgSO_4_  ∗ 7H_2_O, 5.55 mM Dextrose, and 12 mM HEPES. The pH was adjusted to 7.4 at 37°C. The albumin solution was labeled with 0.15 mg/mL Evans Blue. AFC was assessed in living rats as previously described [12, 20, 21] with some modifications. Briefly, at 6 h after injection of LPS or saline by tail vein, the rats were anesthetized with 2% sodium pentobarbital (50 mg/kg, ip). A endotracheal tube was inserted through a tracheotomy. The rats were ventilated with a constant volume ventilator (model TKF-200c; TeLi anesthesia breathing equipment company, Jiangxi, China) with an inspired oxygen fraction of 100%, peak airway pressures of 8–10 cm of H_2_O and positive end expiratory pressure of 3 cm of H_2_O during the baseline period. Following surgery the rats were allowed to stabilize for 10 min. The animals were then placed in the left lateral decubitus position and instillation tubing (16 G Epidural catheter) was gently passed through the tracheotomy tube into the left lung. Then 1.5 mL (5 mL/kg) of the instillate solution with or without CFTR_inh-172_ was instilled at a rate of 0.08 mL/min using a syringe pump. After instillation was complete 0.2 mL of air was injected to achieve complete deposition of all fluid into the alveolar space. The instillate solution remaining in the syringe was collected as the initial sample. Following instillation, the catheter was left in place for the duration of 60 min. The final alveolar sample was collected via the instillation catheter. The concentrations of Evans Blue labeled albumin in the instilled and aspirated solutions were measured by a spectrophotometer at a wavelength of 621 nm. AFC was calculated using the following equation: AFC = 1 − (*C*_*i*_/*C*_*f*_) [12], where *C*_*i*_ is the protein concentration of the instillate before instillation and *C*_*f*_ is the protein concentration of the sample obtained after 60 min of mechanical ventilation. AFC was expressed as a percentage of total instilled volume (%/60 min).

### 2.4. Measurement of IL-6 and TNF-*α* in Lung Tissue Homogenate

Right lung tissue samples were homogenized in 50 mM potassium phosphate buffer (PB, pH 6.0). After three freeze and thaw cycles, with sonication between cycles, the samples were centrifuged at 12,000 rpm for 20 min at 4°C, then aliquoted and stored at −80°C. IL-6 and TNF-*α* were measured by ELISA kits. All procedures were done in accordance with the manufacturer's instructions.

### 2.5. The Haematoxylin-Eosin (H&E) Staining Analysis of the Lung

For histological examination, the right lung tissues were fixed with 4% paraformaldehyde for 24 hours, embedded in paraffin wax, sectioned (5 *μ*m thicknesses). The pulmonary tissue slides were stained with hematoxylin and eosin and were examined using a light microscope (Nikon eclipse 90i, Tokyo, Japan). Analyses of lung tissue slides were carried out by blinded observation to evaluate (a) alveolar congestion; (b) hemorrhage; (c) infiltration or aggregation of neutrophils in airspace or vessel wall; and (d) thickness of alveolar wall/hyaline membrane formation. The results were graded from 0 to 4 for each item, as described previously [22]. The four variables were summed to represent the lung injury score (total score, 0–16).

### 2.6. Immunohistochemistry

After paraffin removal in xylene, the sections were rehydrated and were placed in a pressure cooker with citrate buffer, pH 6.0, for 1 min after reaching boiling temperature to retrieve antigenic sites masked by formalin fixation. After quenching of endogenous peroxidase with 3% of H_2_O_2_ for 15 min, the sections were incubated with rabbit polyclonal antibody to CFTR (1 : 100 dilution) or with the preimmune serum as a negative control stain overnight at 4°C, and subsequently were incubated for 1 h at room temperature with the biotinylated anti-rabbit IgG and peroxidase-conjugated streptavidin, with diaminobenzidine (DAB) as the substrate. The slides were counterstained for 30 seconds with hematoxylin. The slides were observed and photographed with microscopy (Nikon eclipse 90i, Tokyo, Japan).

### 2.7. Isolation and Culture of Primary ATII Cell

ATII cells were isolated from Sprague-Dawley male rats weighing 200–300 g as previously reported with a slight modification [23, 24]. Rats were anesthetized with 2% sodium pentobarbital (50 mg/kg, ip), and then inferior aorta and vena cava were cut. The lungs were perfused with solution A (133 mM NaCl, 5.2 mM KCl, 2.59 mM phosphate buffer, 10.3 mM HEPES buffer, 1 mg/mL glucose, pH 7.4) using a 20-mL syringe fitted with a 23-gauge needle through the right ventricle until the lungs were perfectly turned to white. The lungs were excised with trachea, and were lavaged ten times with solution B (solution A plus 1.89 mM CaCl_2_ and 1.29 mM MgSO_4_) through trachea to remove macrophages, and then completely filled with 0.25% trypsin for 20 min at 37°C. The trachea, main bronchi and large airways were discarded and the lungs were placed onto the sterile petri dish. Each lung was minced into 1-2 mm within 10 min. 5 mL FBS was added to stop trypsin reaction and the tissue suspension was prepared to a final volume of 20 mL by HBSS containing deoxyribonuclease I (250 U/mL). The minced lung tissue was transferred to a 50-mL centrifuge tube in a water bath for 8 min at 37°C. The suspension was filtered through 150 and 75 *μ*m stainless steel meshes and then centrifuged at 1000 rpm for 8 min. The layer containing ATII cells suspended in DMEM containing 10% FBS, 100 U/mL penicillin, 100 mg/mL streptomycin. The cells were plated into 75-cm^2^ flask and incubated for 2-3 h at 37°C in an atmosphere of 95% air-5% CO_2_ to allow fibroblasts to adhere. Nonadherent cells were collected and enriched for ATII cells by differential adherence to IgG-coated plastic dishes for 1 h. At last, unattached cells resuspended in DMEM containing 10% FBS, 100 U/mL penicillin, and 100 mg/mL streptomycin at a density of 2 × 10^6^  cells/mL and cultured on plastic six-well plates. The culture period was limited to 48–72 h so as to minimize dedifferentiation. 

### 2.8. Immunofluorescence

Rat primary ATII cells were grown on coverslips incubated with DMEM stimulated by LPS in the presence or absence of lipoxin A_4_ for 6 h at 37°C. The cells were fixed for 5 min with 4% paraformaldehyde in PBS, and were washed three times with PBS. Fixed cells were permeated with 1% Triton X-100 for 5 min, and were washed three times with PBS. Nonspecific binding of antibodies was prevented by the addition of 5% bovine serum albumin in PBS for 30 min at 37°C. The cells were then incubated with antibody against CFTR (1 : 100) overnight at 4°C. Following three PBS washes, cells were incubated for 2 hours with fluorescein-conjugated goat anti-rabbit IgG (1 : 500), in 5% BSA/PBS at room temperature. After washing three times with PBS, cell nuclei were counter stained with Hoechst (1 : 1000) for 15 min, followed by three PBS washes. Cells were then mounted on a slide and visualized using microscopy.

### 2.9. Western Blotting Analysis

Western blotting analysis from frozen lungs and cells homogenates were performed as described previously [25]. After equal amounts of protein were loaded in each lane and separated by 10% SDS-PAGE, the proteins were transferred to polyvinylidene difluoride membranes. The membranes were blocked for 2 h with 5% skimmed milk, which was also used as primary and secondary antibodies incubation buffer. The primary antibodies were used at dilutions of 1 : 1,000 or 1 : 2,000, and incubated overnight at 4°C. Horseradish peroxidase-conjugated secondary antibodies, which were either goat anti-mouse or goat anti-rabbit, were used at 1 : 2,000 dilution and imaged with the Image Quant LAS 4000 mini (GE Healthcare Bio-Sciences AB, Uppsala, Sweden). Quantification was performed with the AlphaEaseFC software (Alpha Innotech, San Leandro, CA).

### 2.10. Measurement of cAMP in ATII cells

cAMP levels were measured in triplicate by a commercially available enzyme-linked immunosorbent assay (ELISA; from R&D Systems, Minneapolis, MN). The assays were performed according to the manufacturer's protocol.

### 2.11. Statistical Analysis

All values were reported as means ± SEM. Data were analyzed by a one-way ANOVA, followed by a Student Newman-keuls post hoc test and *P* < 0.05 defined as statistically significant. Statistical analysis and graphs were done with GraphPad Prism 5.0 (GraphPad, San Diego, CA).

## 3. Results

### 3.1. The Beneficial Effects of Lipoxin A_**4**_ on LPS-Induced ALI

**Figure 1:**
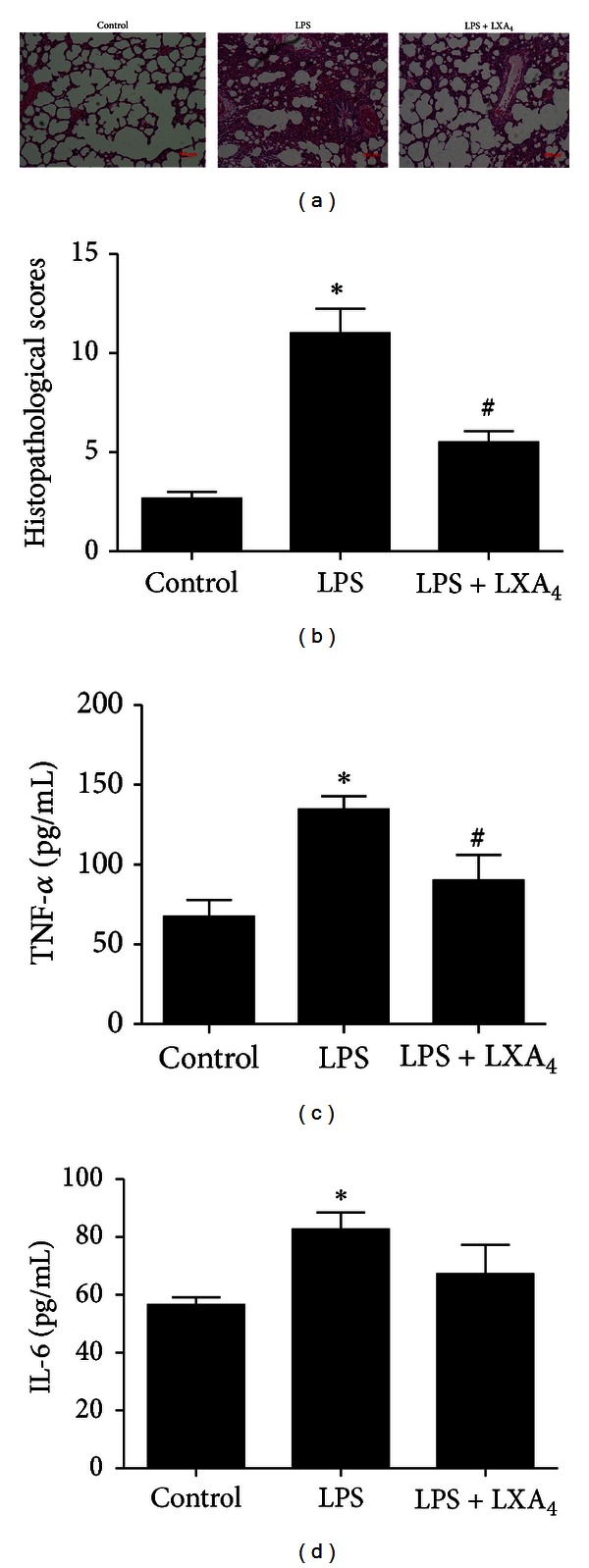
Effects of lipoxin A_4_ on LPS-induced ALI. Lipoxin A_4_ (2 *μ*g/kg) was administered intravenously to Sprague-Dawley (SD) rats 6 h after LPS (20 mL/kg) stimulation through the tail vein, and intratracheal instillation of 5% albumin solution containing Evans Blue-labeled albumin through a tracheostomy to the left lung, and ventilating for 1 hour. The right lung was isolated. The effect of lipoxin A_4_ was assessed by (a) histology in hematoxylin and eosin-stained sections, (b) histopathological scores, (c) the lung tissues homogenate TNF-*α* protein expression, and (d) the lung tissues homogenate IL-6 protein expression. Data were expressed as mean ± SEM for each group. *P < 0.05 versus control group, ^#^ *P* < 0.05 versus LPS group, *n* = 6 for each group. LPS = lipopolysaccharide, LXA_4_ = lipoxin A_4_.

The control group had normal pulmonary histology (Figure 1(a)). In contrast, the lung tissues from the LPS group were significantly damaged with alveolar disarray and severe inflammatory cell infiltration (Figure 1(a)). All indicated that there was ALI in this model. Mild histological changes were observed in the rats lung tissues after treatment with lipoxin A_4_ (Figure 1(a)). Consistent with these histopathological observations, the lung injury score in LPS group was significantly higher than those in control group and LPS + LXA_4_ group (Figure 1(b)). TNF-*α* (Figure 1(c)) and IL-6 (Figure 1(d)) concentration increased significantly in the LPS group compared with control group (*P* < 0.05). This increase in TNF-*α* was significantly (*P* < 0.05) reduced in the lipoxin A_4_ group. Concentration of IL-6 in lipoxin A_4_ group (67.24 ± 24.56) was lower than LPS group (82.74 ± 14.04), although the different was not significant (*P* > 0.05). 

### 3.2. The Effect of Lipoxin A_**4**_ on LPS-Stimulated AFC

Different concentrations of LPS were used in the experiments. We found the effect of 10 mg/kg LPS on AFC is unstable in rats. However, 15 mg/kg LPS could inhibit AFC, and reached the significant effect at 20 mg/kg, so we chose 20 mg/kg LPS in our experiments. We also have performed different concentrations of lipoxin A_4_ including 1 *μ*g/kg, 1.5 *μ*g/kg, 2 *μ*g/kg, 2.5 *μ*g/kg, 3 *μ*g/kg in our experiments. We found that 1 *μ*g/kg lipoxin A_4_ had no influence on improving the AFC reduced by LPS; 1.5 *μ*g/kg lipoxin A_4_ improved the AFC reduced by LPS, and reached the maximal effect at 2 *μ*g/kg, the effect of lipoxin A_4_ was similar between 2 *μ*g/kg and 2.5 *μ*g/kg, 3 *μ*g/kg, so 2 *μ*g/kg lipoxin A_4_ was chosen in our experiments (data not shown). 

**Figure 2:**
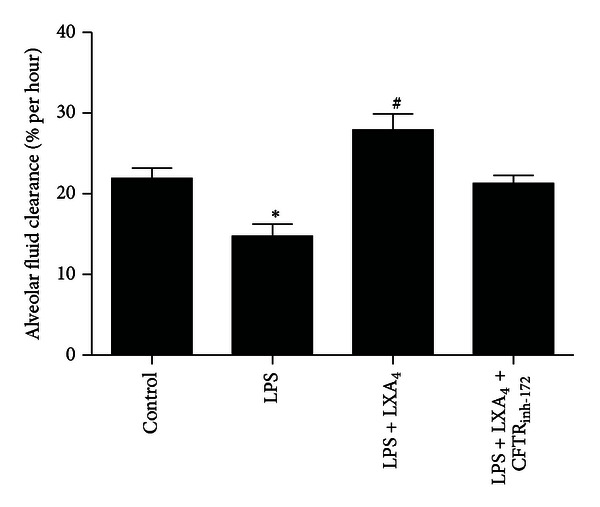
Effects of lipoxin A_4_ on AFC in LPS-induced ALI. Lipoxin A_4_ (2 *μ*g/kg) was administered intravenously to Sprague-Dawley (SD) rats 6 h after LPS (20 mL/kg) stimulation through the tail vein, and intratracheal instillation of 5% albumin solution containing Evans Blue-labeled albumin through a tracheostomy to the left lung, ventilating for 1 hour. CFTR_inh-172_ was instilled with albumin solution. Data were expressed as mean ± SEM for each group. *P < 0.05 versus control group, ^#^ *P* < 0.05 versus LPS group, *n* = 8 for each group. LPS = lipopolysaccharide, LXA_4_ = lipoxin A_4_.

AFC was found to be markedly decreased in the LPS (20 mg/kg) group as compared with control group (*P* < 0.05). The decrease was significantly (*P* < 0.05) reduced in the lipoxin A_4_ (2 *μ*g/kg) group (Figure 2). Next, we tried to block chloride ion channel using CFTR_inh-172_ (1 uM), a specific inhibitor of CFTR. We found that the effect of lipoxin A_4_ on AFC was abolished by the treatment with CFTR_inh-172_ (Figure 2).

### 3.3. The Effect of Lipoxin A_**4**_ on Protein Expression of CFTR In Vivo and In Vitro Experiments

**Figure 3:**
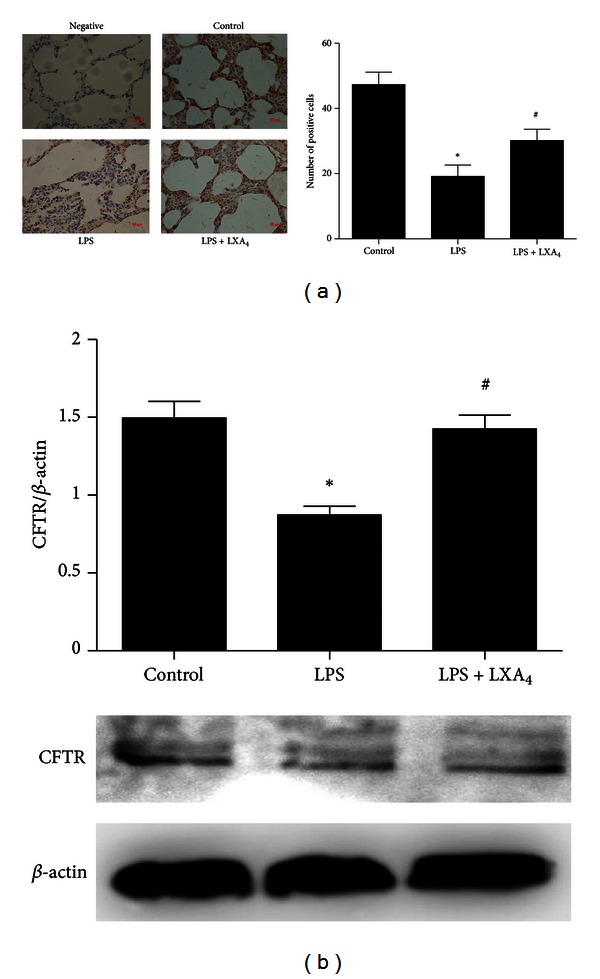
Effects of lipoxin A_4_ on protein expression of CFTR *in vivo.* Lipoxin A_4_ (2 *μ*g/kg) was administered intravenously to Sprague-Dawley (SD) rats 6 h after LPS (20 mL/kg) stimulation through the tail vein, and intratracheal instillation of 5% albumin solution containing Evans Blue-labeled albumin through a tracheostomy to the left lung, ventilating for 1 hour. The right lung was isolated. (a) The protein expression of CFTR was assessed by immunohistochemistry. Immunopositive cells were counted in five randomly selected nonoverlapping fields of three separately immunostained lung sections per group. (b) The right lung was also homogenized for western blotting. Data were expressed as mean ± SEM for each group. *P < 0.05 versus control group, ^#^ *P* < 0.05 versus LPS group. LPS = lipopolysaccharide, LXA_4_ = lipoxin A_4_, CFTR = cystic fibrosis transmembrane conductance regulator.

**Figure 4:**
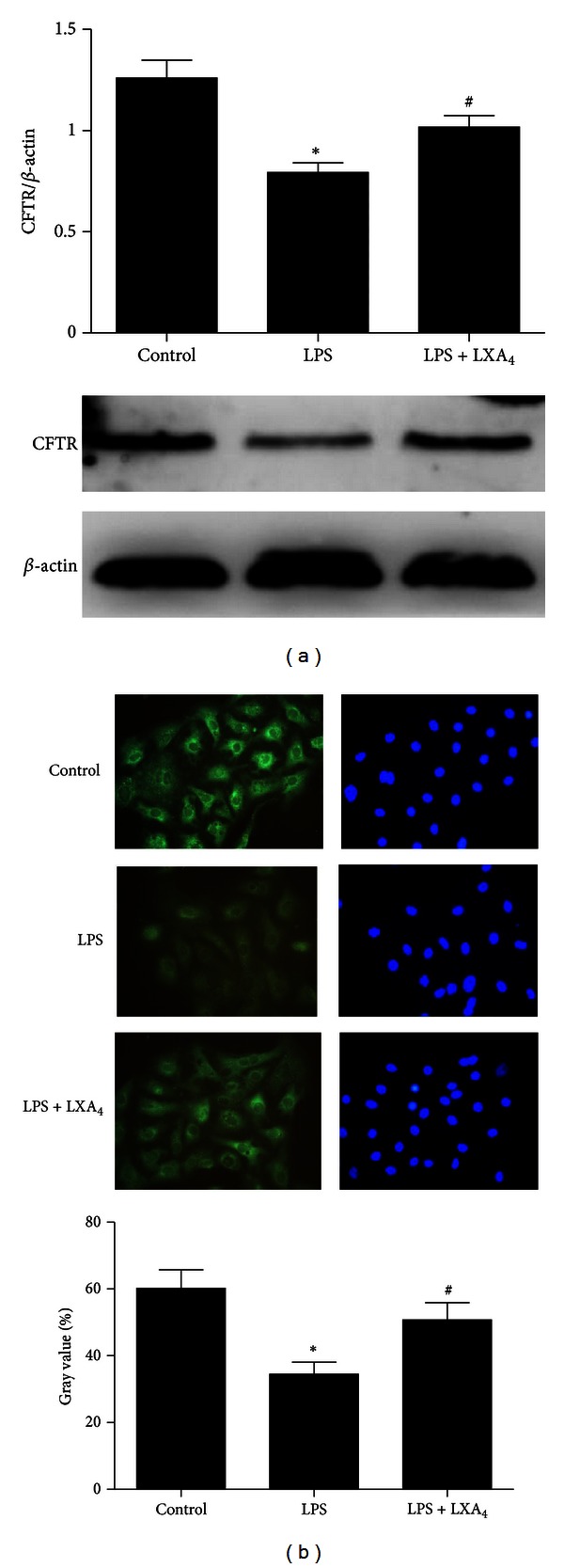
Effects of lipoxin A_4_ on protein expression of CFTR in primary ATII cells. The cells were treated with lipoxin A_4_ (100 nM) in the presence of LPS (1 *μ*g/mL) for 6 hours. (a) After incubation, the cells were harvested, sonicated and CFTR protein detected by western blotting. (b) The protein expression of CFTR also was detected by immunofluorescence assay. Data were expressed as mean ± SEM for each group. *P < 0.05 versus control group, ^#^ *P* < 0.05 versus LPS group, *n* = 4 for each group. LPS = lipopolysaccharide, LXA_4_ = lipoxin A_4_, CFTR = cystic fibrosis transmembrane conductance regulator.

*In vivo*, the expression of CFTR protein in the lung tissue homogenate was detected by immunohistochemistry assay, which was decreased by LPS stimulation, but enhanced by lipoxin A_4_ treatment (Figure 3(a)). The change in western blotting analysis was similar to the result in immunohistochemistry assay (Figure 3(b)). CFTR protein expression was significantly increased in the lipoxin A_4_ treatment group compared with the LPS group in rat primary ATII cells (Figure 4(a)). Result in immunofluorescence assay was consistent with that in western blotting analysis (Figure 4(b)).

### 3.4. LPS Decreases Protein Expression of CFTR via PI3K/Akt Signaling Pathway in Primary ATII Cells

**Figure 5:**
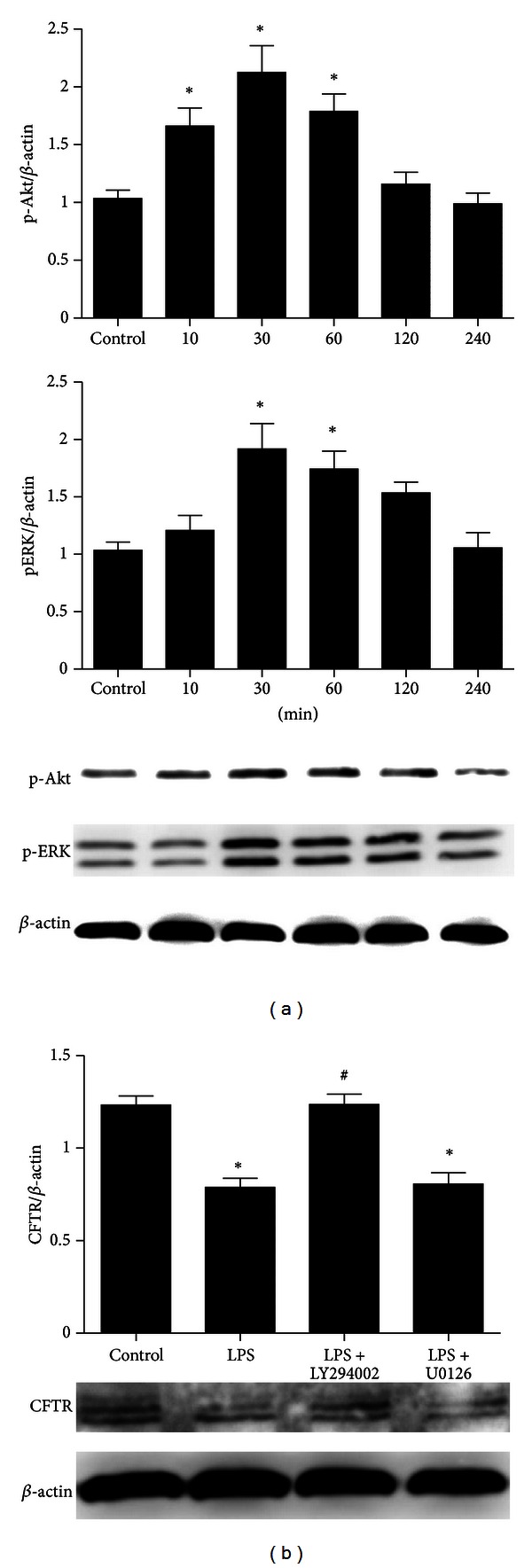
LPS decreases protein expression of CFTR via PI3K/Akt signaling pathway in ATII cells. The cells were incubated with LPS (1 *μ*g/mL) for 10, 30, 60, 120, and 240 min. After incubation, the cells were harvested and sonicated. (a) Phosphorylation of Akt and ERK in the cell lysates were assessed by western blotting. (b) The rat primary ATII cells were pretreated with LY294002 (10 *μ*M, PI3K/Akt inhibitor) or U0126 (20 *μ*M, ERK inhibitor) for 30 min and then incubated with LPS (1 *μ*g/mL) for 6 h. After incubation, the cells were harvested, sonicated and CFTR protein detected by western blotting. Data were expressed as mean ± SEM for each group. *P < 0.05 versus control group, ^#^ *P* < 0.05 versus LPS group, *n* = 4 for each group. LPS = lipopolysaccharide, CFTR = cystic fibrosis transmembrane conductance regulator.

To investigate which signaling pathway was involved in the regulation of LPS on CFTR protein expression, firstly, we tested the phosphorylation of Akt and ERK after rat primary ATII cells were stimulated by LPS. The phosphorylation of ERK and Akt reached a peak within 30 min (Figure 5(a)). Secondly, ATII cells were pretreated with PI3K/Akt kinase inhibitor (LY294002) and ERK inhibitor (U0126) for 30 min. Only the addition of LY294002 abrogated LPS-induced downregulation of CFTR protein expression (Figure 5(b)).

### 3.5. Lipoxin A_**4**_ Suppresses LPS-Stimulated Phosphorylation of Akt

**Figure 6:**
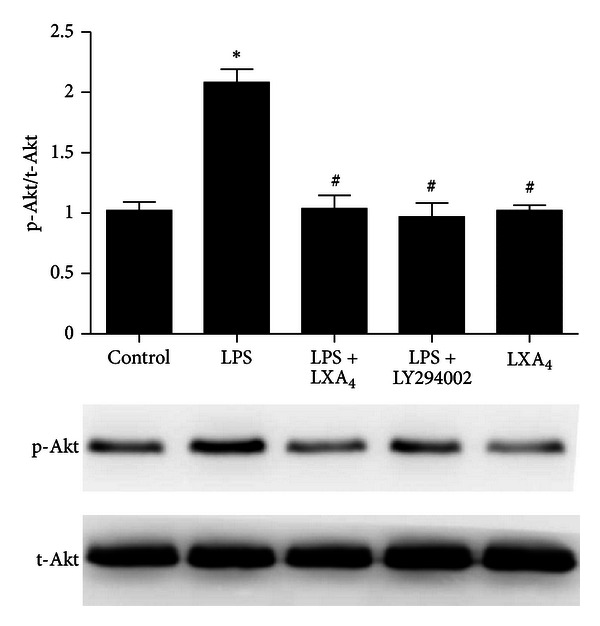
Lipoxin A_4_ reverses LPS-induced phosphorylation of Akt. ATII cells were treated with LY294002 (10 *μ*M, PI3K/Akt inhibitor) or lipoxin A_4_ (100 nM) in the presence of LPS (1 *μ*g/mL) for 30 min. After incubation, the cells were harvested and sonicated. Phosphorylation of Akt in the cell lysates was assessed by western blotting. Data were expressed as mean ± SEM for each group. *P < 0.05 versus control group, ^#^ *P* < 0.05 versus LPS group, *n* = 4 for each group. LPS = lipopolysaccharide, LXA_4_ = lipoxin A_4_.

Phosphorylation of Akt was significantly reduced in lipoxin A_4_ treatment group compared with LPS group. Phosphorylation of Akt was also decreased in LY294002 administrated cells (Figure 6). The experiment was repeated four times with similar results.

### 3.6. The Effect of Lipoxin A_** 4**_ on Intracellular cAMP in LPS-Stimulated ATII Cells

**Figure 7:**
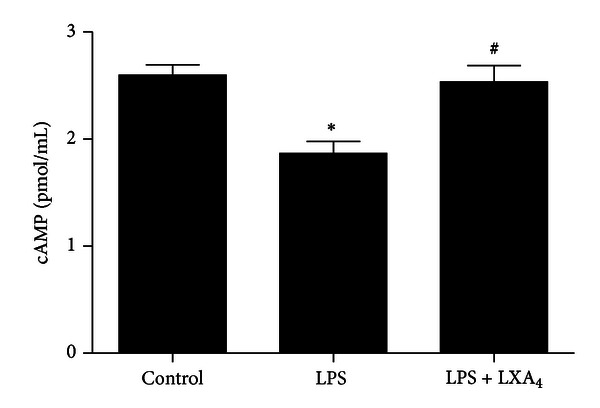
The effect of lipoxin A_4_ on cAMP in ATII cells. The cells were treated with lipoxin A_4_ (100 nM) in the presence of LPS (1 *μ*g/mL) for 1 hour. After incubation, the cells were harvested and intracellular cAMP was detected by Elisa Kits. Data were expressed as mean ± SEM for each group. *P < 0.05 versus control group, ^#^ *P* < 0.05 versus LPS group. *n* = 6. LPS = lipopolysaccharide, LXA_4_ = lipoxin A_4_.

We measured the intracellular cAMP levels in ATII cells after 1 h of exposure to LPS or LPS plus lipoxin A_4_ (Figure 7). cAMP level was decreased in LPS group compared with control group (*P* < 0.05), but LPS + LXA_4_ group increased the cAMP level compared with LPS group (*P* < 0.05).

## 4. Discussion

ALI and its more severe form, the acute respiratory distress syndrome (ARDS), are relatively common syndromes in critically ill patients associated with high morbidity and mortality [26]. No specific therapy is currently available to modulate this inflammatory response and protect the lung. Experimental strategies to block prophlogistic mediators have not proven successful [27] because of the multiple, redundant pathways that initiate inflammation. These strategies also decrease the host's ability to adequately deal with infection, given that the innate inflammatory response is a beneficial defensive event [28]. Traditionally, it was argued that pro-inflammatory mediator catabolism was sufficient for inflammation to switch off, with the subsequent responses ending passively. New evidence indicates that resolution of inflammation and the return to homeostasis is not a passive, but an actively regulated process [29]. Specifically in ALI, resolution is characterized by clearance of PMN from the lung and reabsorption of alveolar fluid [30]. Within the resolution phase, endogenous proresolving lipid mediators, such as lipoxins, can counter-regulate host inflammation responses and promote resolution [31]. Taking these data together, our purpose was to evaluate whether lipoxin A_4_ have protective action against LPS-induced ALI and promote reabsorption of alveolar fluid.

Our data clearly demonstrated that treatment of rats with lipoxin A_4_ significantly inhibited IL-6, TNF-*α* production and increased AFC with the outcome of decreased pulmonary edema in lung tissue. The inhibitor of CFTR was able to abolish the beneficial effects of lipoxin A_4_. Furthermore, we demonstrated that treatment with lipoxin A_4_ upregulated the CFTR protein expression *in vivo* and primary ATII cells. Finally, our data provided evidence that LPS decreased CFTR protein expression via PI3K/Akt signaling pathway and the lipoxin A_4_ can suppress LPS-stimulated phosphorylation of Akt. 

LPS stimulates macrophages, neutrophils, and other immune cells to produce different mediators including cytokines such as TNF-*α*, IL-6 that recruits polymorphonuclear neutrophils into the injured site [32]. In addition, activated neutrophils transmigrate across the endothelial surface into lung by release of reactive oxygen species, resulting in alveolar capillary barrier leakage, interstitial and alveolar edema [33]. Accumulating data indicated that upregulation of CFTR increased AFC [7, 34]. The lack of functional CFTR in ΔF508 mice could limit their capacity to remove alveolar edema [8]. Transforming growth factor *β*_1_ inhibited AFC via downregulation of CFTR protein expression [35]. 

 CFTR protein expression is regulated by a complex network of signaling pathways, including the Akt and ERK pathways [35, 36]. Akt is a key mediator of signal transduction in protein synthesis and is a downstream kinase of PI3K [37]. The PI3K/Akt pathway has been shown to mediate TGF-*β*_1_ induced decrease of CFTR protein expression in primary ATII cells [35]. It is known that LPS can bind to toll-like receptor 4 (TLR4), which acts as receptor that activates downstream signaling pathways including PI3K/Akt [38]. The present study revealed that culture of primary ATII cells with LPS resulted in a rapid phosphorylation of Akt and ERK. The phosphorylation of ERK and Akt reached a peak within 30 min. Only the addition of LY294002 (PI3K/Akt inhibitor) abrogated LPS-induced down-regulation of CFTR protein expression. These findings suggest that LPS inhibits CFTR protein expression via activation of the PI3K/Akt pathway.

We found that lipoxin A_4_ downregulated the LPS-stimulated phosphorylated Akt in primary ATII cells. Previous studies have showed that lipoxin A_4_ inhibited connective tissue growth factor (CTGF)-induced proliferation of human lung fibroblasts via down-regulation of PI3K/Akt [39]. Aspirin-triggered lipoxin A_4_ also inhibited myeloperoxidase (MPO) suppression of neutrophil apoptosis via down-regulation of PI3K/Akt [40]. In the current study, the effects of lipoxin A_4_ mimicked the inhibition of PI3K/Akt with LY294002, suggesting that the PI3K/Akt pathway also regulates the effect of lipoxin A_4_ on LPS induced decrease of CFTR protein expression. 

cAMP is a ubiquitous second messenger regulating a majority of intracellular functions. Extracellular signals interact with GPCRs to activate adenylate cyclase and increase the intracellular cAMP levels. Experiments on duodenal epithelial cells suggested that cAMP not only increased the activity of CFTR but also shifted CFTR proteins to the plasma [41]. Previous study reported that pretreatment of the bronchial epithelial cells with either MDL hydrochloride (adenylate cyclase inhibitor) or (Rp)-cAMP (cAMP-dependent protein kinase inhibitor) inhibited the Ca^2+^ response to lipoxin A_4_. Pertussis toxin treatment completely abolished the Ca^2+^ response induced by lipoxin A_4_ in 16HBE14o^−^ cells [42]. These found suggested that lipoxin A_4_ up-regulated CFTR expression may through the cAMP pathway.

In conclusion, this study demonstrates that administration of lipoxin A_4_ systemically increases AFC in LPS injured rat lungs. We also demonstrate that augmented AFC is associated with enhanced CFTR protein expression after treatment with lipoxin A_4_. The mechanism may be through PI3K/Akt signaling pathway. Our findings reveal a novel mechanism for pulmonary edema fluid reabsorption and lipoxin A_4_ may provide new opportunities to design “reabsorption targeted” therapies with high degree of precision in controlling ALI/ARDS.
